# Epidemiology of Toxoplasma and CMV serology and of GBS colonization in pregnancy and neonatal outcome in a Sicilian population

**DOI:** 10.1186/1824-7288-40-23

**Published:** 2014-02-22

**Authors:** Giuseppe Puccio, Cinzia Cajozzo, Laura Antonella Canduscio, Lucia Cino, Amelia Romano, Maria Gabriella Schimmenti, Mario Giuffrè, Giovanni Corsello

**Affiliations:** 1 Department of Sciences for Health Promotion and Mother and Child Care, University of Palermo, Palermo, Italy; 2 U.O. Malattie Infettive Pediatriche, P.O. “G. Di Cristina”, ARNAS Civico – Di Cristina – Benfratelli, Palermo, Italy

**Keywords:** Epidemiology, Toxoplasma, CMV, GBS, Intrauterine infection

## Abstract

**Background:**

Aim of our study is to analyze the immunological status in pregnancy for two main TORCH agents, Toxoplasma and Cytomegalovirus (CMV), and the results of group B streptococcus (GBS) screening, assessing the risk for congenital infection in a population from Palermo, Italy.

**Methods:**

We retrospectively analyzed the medical records of all inborn live newborns who were born in our division during 2012, gathering information about the mother, the pregnancy and neonatal hospitalization at birth. Whenever data were available, we categorized the serologic status of the mothers for Toxoplasma and CMV. We also considered the results of rectal and vaginal swabs for GBS. We compared the results in Italian and immigrant mothers. The neonatal outcome was evaluated in all cases at risk.

**Results:**

Prevalence of anti-Toxo IgG antibodies was 17.97%, and was significantly higher in immigrant women (30% vs 16.4% in Italian women; p = 0.0008). Prevalence of anti-CMV IgG antibodies was 65.87%. Again, it was significantly higher in immigrant women (91.4% vs 62.5%, p = 3.31e-08). We compared those data with a previous study performed in our hospital in 2005–2006, and found that the prevalence of anti-Toxoplasma and anti-CMV antibodies in our population has remained stable, both in the immigrant and in the local population. Seroconversion rates and neonatal infections were rare: no seroconversions were observed for Toxoplasma, 4 seroconversions for CMV. One neonatal Toxoplasma infection and two neonatal CMV infections were documented. In some cases with dubious patterns or probable persistence of IgM, we performed additional tests and follow-up. Vaginal and rectal swabs were positive for GBS in 7.98% of cases, with no significant difference between the Italian and the immigrant population. No GBS neonatal sepsis was documented.

**Conclusions:**

The prevalence of Toxoplasma IgG antibodies in pregnant women was low in our population, if compared with European countries and with other parts of Italy, and is significantly higher in immigrant women. The prevalence of CMV IgG antibodies was intermediate if compared to other countries, and it was higher in immigrant women. GBS positivity was low, and comparable in Italian and immigrant mothers. Neonatal infection was rare for all these agents.

## Background

Neonatal infections, both congenital and nosocomially acquired, are a major issue of concern and interest for all neonatal units who therefore develop strategies for monitoring and prevention of vertical (mother to fetus) and horizontal (environment to newborn) transmission.

Evaluation of the status of pregnant women for those infections that can affect the fetus is standard routine. Toxoplasmosis [1] and CMV [2] infections, in our context, are checked in most pregnancies, as will be explained in the “Methods” section, and a recto-vaginal swab for GBS has become an usual practice too, aimed to prevention of early neonatal sepsis by intrapartum antibiotic prophylaxis [3].

Many problems, however, still arise in the clinical practice. The assessment of the immunological status of the mothers is not always simple, and may require second level tests such as IgG avidity, Immunoblotting, PCR, especially when the scenario is not clear at first evaluation (for example, when both IgG and IgM antibodies are present at first test, and the timing of infection is not clear). The same can be said for the evaluation of the neonate who is at risk of congenital infection. Moreover, the follow-up of the mother during pregnancy is not always optimal, consistent and complete.

The epidemiology of the immune status for Toxoplasma and CMV infections in women of reproductive age is certainly an important variable in the clinical approach to these problems. The prevalence of IgG antibodies for those agents in pregnant women varies in different countries and ethnicities, and according to socio-economical factors like social class, education, job. Data available in the scientific literature are limited, and need to be periodically updated.

For GBS, much has been done to understand its epidemiology, its role as an agent of neonatal sepsis and the best strategies to prevent such an outcome. The Centers for Disease Control (CDC), an USA federal agency under the Department of Health and Human Services, have recommended the practice of universal screening of pregnant women in the third trimester and intrapartum antibiotic prophylaxis in positive cases. As a consequence of that, neonatal sepsis has been efficiently controlled in USA, with a reduction in its prevalence from 1.7/1000 liveborn babies at the beginning of the ’90s to 0.34-0.37/1000 liveborn babies in the last few years [3].

The aim of our study is to provide some data about the immunological status in pregnant women for the two main TORCH agents, Toxoplasma and CMV, and the results of GBS screening in the third trimester, in order to assess the risk for congenital infection in the newborn in a population from the city and province of Palermo, Sicily (Italy).

## Methods

We retrospectively analyzed the medical records of all live babies who were born in the Department of Sciences for Health Promotion and Mother and Child Care of the University of Palermo from 01/01/2012 to 31/12/2012. For each of them, we recorded the pertinent information about the mother, the pregnancy and neonatal hospitalization at birth, and in particular:

Mother and pregnancy:

• Mother’s age at delivery and mother’s nationality and country of birth;

• Mother’s antibody status for Toxoplasma and CMV, as ascertained during pregnancy;

• The results of rectal and vaginal swab test for GBS in the last trimester of pregnancy;

• Delivery type: vaginal, elective Cesarean Section or emergency Cesarean Section;

• Maternal Premature Rupture Of Membranes (PROM) (>18 h before delivery);

Newborn:

• Sex;

• Gestational age;

• Apgar score at 1 and 5 minutes;

• Weight, length and cranial circumference at birth, and respective percentiles;

• First blood cell count after birth (if available);

• Highest C Reactive Protein (CRP) value (if available) during hospitalization;

• Neurological, respiratory or alimentary pathologies during hospitalization;

• Eventual admission to Neonatal Intensive Care Unit (NICU) for neonatal pathologies;

• Eventual intravenous antibiotic therapy;

Statistical analysis was performed by the open source statistical software R [4].

### Population description

899 live babies were born in our hospital in 2012 (there were 8 stillborns in the same period, which were not considered in this study). The total number of deliveries was 881, with 16 twin births and 1 triplet birth. The medical records were not available for 8 newborns, so our analysis was performed on 891 newborns. Tables 1 and 2 sum up the general features of our population. Screening for toxoplasma, CMV and GBS during pregnancy is usually performed routinely, but its execution depends on many variables: the gynecologist who is in care of the mother during pregnancy (usually a private gynecologist, or some hospital service), the timing of first medical control during pregnancy, and the compliance of the mother herself. Usually, for toxoplasma and CMV a first evaluation is performed as soon as possible after the diagnosis of pregnancy, and then repeated, possibly monthly, in all negative women. Women with the presence of IgG and absence of IgM antibodies at first evaluation don’t usually undergo any other tests, but there are some exceptions. If IgG and IgM antibodies are both present at first test, second level exams are usually performed (IgA, IgG avidity, Immunoblotting) to assess, if possible, the timing of infection. However, there are many variations of behavior in individual cases. For our study, we considered all the cases where at least one routine test for toxoplasma and/or CMV antibodies had been performed during pregnancy. According to the results of all tests performed in each individual case, we classified the mothers in the following categories:

a) Immune mother: Presence of specific IgG antibodies at first test during pregnancy, absence of IgM antibodies.

b) Non immune mother: Absence of specific IgG and IgM antibodies throughout pregnancy.

c) Seroconversion during pregnancy: appearance of specific IgG and/or IgM antibodies during pregnancy, after a previous negative test.

d) Presence of IgM antibodies: at least in one test during pregnancy, with positive IgG antibodies in the absence of any evidence of seroconversion,

e) Dubious: inconsistent patterns, possible lab errors.

**Table 1:** General features of our population (categorical data)

			
Maternal nationality	Italian	784	87.89%
	Not italian	108	12.11%
Maternal job	Housewife	505	66.01%
	Employee	170	22.22%
	Craftwoman/tradewoman	25	3.27%
	Professional	65	8.50%
Delivery type	Vaginal	414	46.67%
	Elective caesarean section	329	37.09%
	Emergency caesarian section	144	16.23%
Sex of the newborn	F	455	51.07%
	M	436	48.93%

**Table 2:** General features of our population (numerical data)

	**Mean**	**SD**	**Median**	**Range**
Mother’s age (year)	30.46	5.91	30.64	15.57 – 51.49
Gestational age (week)	39	1.8	39.1	26.1 – 42.3
Weight at birth (g)	3174.11	539.14	3220	630 - 4760
Lenght at birth (cm)	49.1	2.6	49.5	31 – 55.4
Cranial circumference (cm)	33.8	1.6	34	23.5 – 39.6

We also considered the results of rectal and vaginal swabs for GBS in the third trimester of pregnancy. This is usually performed routinely according to the indications of the CDC [3], but again there are many variations of practice according to individual gynecologists and/or mother’s compliance. Moreover, the systematic practice of this test is relatively recent in our context, and not yet universally implemented.

A further analysis was performed comparing Italian mothers and immigrant mothers, to detect possible differences between the two groups. Immigrant mothers were defined as those who were not born in Italy and were not Italian citizens at birth. In our population, immigrant mothers were 10.54%. Most frequent countries of birth were Romania (20.65%), Bangladesh (16.30%), Ghana (8.70%) and Sri Lanka (7.61%).

## Results

Anti-Toxoplasma antibodies were tested during pregnancy, at least once, in 846 cases (94.84%).

IgG antibodies were present in 152/846 pregnant women, which corresponds to a prevalence of 17.97% (95% CI = 15.53% - 20.70%). In 3 cases IgM antibodies were also present at least in one test during pregnancy, in the absence of any evidence of seroconversion. In two cases the results of the tests were dubious or inconsistent. No case with a documented seroconversion was found.

There was no significant difference in maternal age between the two groups of immune and non immune mothers (median 30.9 vs 30.61 years; p = 0.21). Prevalence of anti-Toxo antibodies was not significantly related to maternal job: 17.9% in housewives, 14.2% in employees, 24% in craftswomen and tradeswomen, 17.5% in professionals, p = 0.56).

The prevalence of anti-Toxo antibodies was significantly higher in immigrant mothers (30% vs 16.4%; p = 0.0008, chi square test for independence). The ratio of the prevalence of immune serology in immigrant mothers vs local mothers was therefore 1.83 (95% CI 1.3 – 2.58).

All 3 newborns from mothers that presented IgM antibodies during pregnancy were referred to the Paediatric Infectious Diseases Unit for follow-up. One of them was found to be infected, and was successfully treated for about one year. In another one, no neonatal infection was found. The third neonate was lost to follow-up before his condition could be definitely assessed. In the two dubious cases, the pattern of maternal tests showed no real risk for neonatal infection, and was easily explained as the result of lab errors. Therefore, no neonatal follow-up was performed.

Anti-CMV antibodies were tested during pregnancy, at least once, in 797 cases (89.2%).

IgG antibodies were present in 525/797 pregnant women. That corresponds to a prevalence of 65.87% (95% CI = 62.51% - 69.08%). Only in 4 cases (0.5%) a seroconversion was observed during pregnancy, while in 8 cases (1%) IgM antibodies were present together with IgG at least in one test during pregnancy, in the absence of any evidence of seroconversion.

In 10 cases (1.25%) the results of the tests were dubious or inconsistent.

In all cases of seroconversion or presence of IgM antibodies and in most dubious results a test for CMV DNA in urine was effected in the newborn, in the first week of life, and it was positive only in two cases, one of the 4 babies from mothers with seroconversion and one of the 8 babies from mothers with IgM antibodies during pregnancy. Those two cases of neonatal infection were referred to the Paediatric Infectious Diseases Unit for follow-up, and after further clinical evaluation they were considered completely asymptomatic, and they received no therapy. Clinical follow-up is ongoing (Table 3).

**Table 3:** Results of CMV DNA tests in the newborn in cases at risk for neonatal infection

**Type of risk**	**N**	**CMV test in urines**	**CMV +**
Maternal seroconversion	4	4	1
Presence of IgM	8	8	1
Dubious serology	10	8	0

There was no significant difference in maternal age between the two groups of CMV immune and non immune mothers (median 30.6 vs 31.18; p = 0.73).

Prevalence of anti-CMV antibodies was weakly related to maternal job, and we observed a slightly lower prevalence in professionals (52.5%, vs 67.9% in housewives, 59.6% in employees, 70.8% in craftswomen and tradeswomen, p = 0.049, chi square test for independence).

The prevalence of anti-CMV antibodies was significantly higher in immigrant mothers (91.4% vs 62.5%; (p = 3.31e-08, chi square test for independence). The ratio of the prevalence of immune serology in immigrant mothers vs local mothers was therefore 1.46 (95% CI 1.34 – 1.59).

Rectal-vaginal swab for GBS was performed in 589 cases (66.03%) and it was positive in 47 cases (7.98% of the tests, 95% CI = 6.05% - 10.45%). Intrapartum antibiotic prophylaxis was performed in 28/47 positive cases (60%), and in 28/542 negative cases (5.2%), usually for the presence of clinical risk factors. Most cases where the prophylaxis was not performed were cesarean sections with intact membranes, but in a few cases prophylaxis was simply omitted, probably for lack of the correct information at the time of delivery. However, no GBS neonatal sepsis was demonstrated in our population.

There was no significant difference in maternal age between the two groups of positive and negative mothers (median 29.4% vs 30.9%; p = 0.38).

Prevalence of GBS positivity was not significantly related to maternal job (p = 0.12).

Unlike what we observed for Toxoplasma and CMV, the prevalence of positivity for GBS was not significantly higher in immigrant mothers (7.6% in Italian and 10.6% in immigrant mothers, p = 0.40).

## Discussion

Perinatal infections are a major cause of morbidity and mortality in the neonatal age in developed countries. Despite the increasing role of horizontally transmitted nosocomial infections [5,6], especially in infants who require prolonged hospitalization because of underlining risk factors (prematurity, IUGR, congenital malformations) [7,8], vertically transmitted perinatal infections are still frequent and responsible for short and long term sequelae. Neonatal Units must therefore pay attention both to evaluate and limit the circulation of outbreaks of nosocomial pathogens [9] and also to identify the risk of mother to fetus/newborn transmission of well known dangerous pathogens, such as Toxoplasma, CMV and Group B Streptococcus.

Our data provide some insights about the immune status for Toxoplasma and CMV of pregnant women in our area (city and province of Palermo, Sicily).

**Figure 1:**
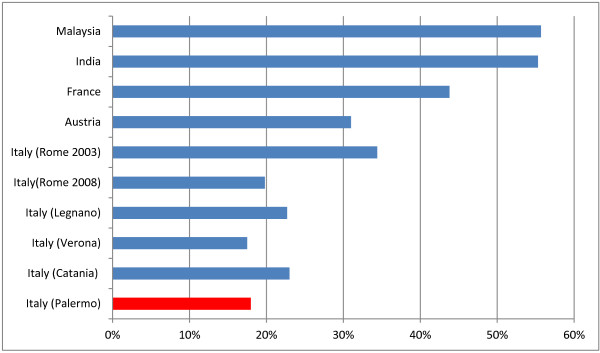
Prevalence of Toxoplasma IgGs in women of reproductive age or pregnant in various countries.

The prevalence of anti-Toxo IgG in our sample is 17.97%, and it is roughly comparable to data from other Italian regions, confirming the medium-low prevalence of Toxoplasma infection in Italy if compared with high prevalence countries (such as France [10] or Austria [11], Latin America and South-east Asia). Our value is indeed one of the lowest reported in Italy, very similar to data from Verona (17.5%), and definitely lower than values reported from Rome (19.8% - 34.4%) or Lombardy (22.7%). The only other reported value from Sicily (Catania) is slightly higher too (23%) [1] (Figure 1).

Our data also show that prevalence of anti-Toxo IgG antibodies is significantly higher in immigrant women (30% vs 16.4% in Italian women). Similar differences have been observed in other countries, for example in USA, where the general prevalence is 11%, with 7.7% for US born and 28.1% for foreign born women of child-bearing age [1].

Obviously, all these differences can be variously explained taking into account different alimentary habits and styles, and differences in social and sanitary levels between local and immigrant populations.

The prevalence of anti-CMV antibodies in our pregnant women was medium-high (65.87%), comparable to other Italian data (Legnano, Lombardy: 68.3%) [12].

**Figure 2:**
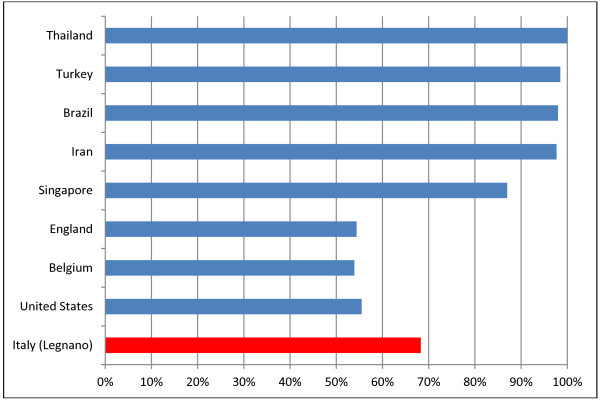
Prevalence of CMV IgGs in women of reproductive age or pregnant in various countries.

According to data from scientific literature, the prevalence of CMV infection is higher in developing countries and in low socioeconomic areas [2] (Iran 97.69% [13]; Brazil 98%) [14], while in industrial countries it is definitely lower (Belgium 53.9% [15]; England 54.4% [16], USA 55.5%) [17] (Figure 2).

In these countries, where the data are available, a significant difference in prevalence can be observed between different types of population: in England, the prevalence was very high in Asian women (88.2%), and lowest in the white population (45.9%) [16]; a similar pattern was observed in the USA (77.2% in black females, 78.2% in Hispanic females, 45.2% in white females [17]). In our sample, too, the prevalence is higher in immigrant women (91.4% vs 62.5% in Italian women).

Again, differences between social and sanitary levels in immigrant women, both in their originary countries and in our country, and in Italian women are the most likely explanation for the observed data.

**Figure 3:**
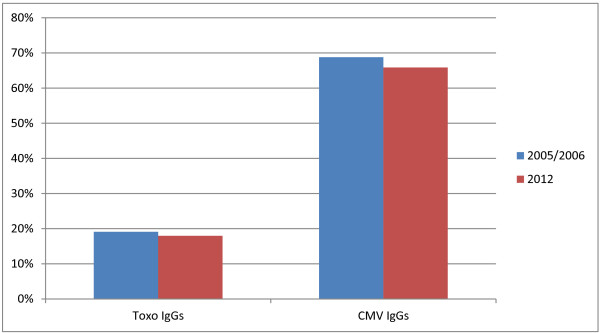
Toxoplasma and CMV seroprevalence in 2005/2006 and in 2012.

We had already performed a similar study in 2005–2006 in our hospital [18]. Therefore, we were interested in comparing our new data with those of the previous study, to verify and confirm those results and/or detect possible epidemiological variations after a few years. As shown in Figure 3, the prevalence of anti-Toxoplasma and anti-CMV antibodies in our population has remained quite stable in the last few years, with only a slight reduction in both cases.

**Figure 4:**
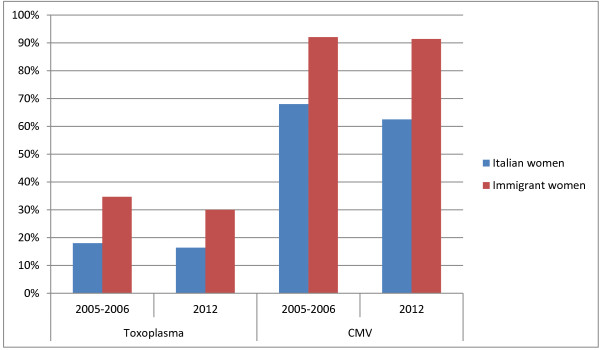
Toxoplasma and CMV seroprevalence in 2005/2006 and in 2012: differences between Italian women and immigrant women.

The same is true if we compare data about immigrant and Italian women from the two studies, as shown in Figure 4.

Vaginal and rectal swabs in our population of pregnant women were positive for GBS in 7.98% of cases; this value is slightly lower than the national average, which is about 10-20% [19]: in North Eastern Italy it is reported as 17.9% [20], in Perugia 11.3% [21], in Friuli Venezia Giulia 19.7% [22].

**Figure 5:**
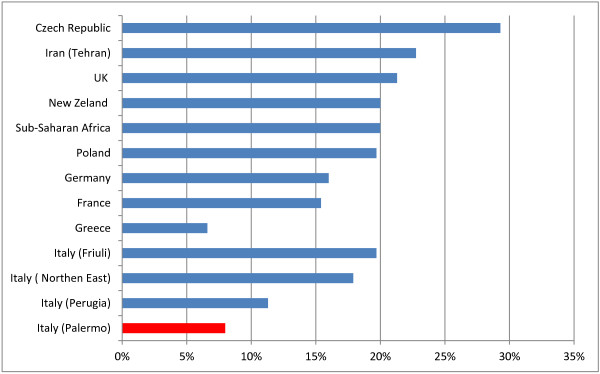
GBS: maternal colonization rates in various countries.

In European countries the prevalence of GBS carriers among pregnant women varies between 6.5% and 36% with one third of studies reporting rates of 20% or greater. Maternal GBS colonization rates in different European countries appear to be similar to those reported in other industrialized countries, such as the US (10-30%), Canada (11–19.5%) and New Zeland (20%) (Figure 5) [23].

We could not find a significant difference between Italian and immigrant women for GBS prevalence, although a slightly lower value was observed in local mothers, which is in line with what is reported in one USA study (13.7% in white women, vs 21.2% in black and 20.9% in Hispanic).

Interestingly, anti-Toxoplasma antibodies were performed in almost all pregnancies (94.84%), while anti-CMV antibodies were performed in 89.2% of cases; this is perhaps a sign that gynecologists are, in some measure, more “aware” of Toxoplasma rather than CMV as a possible problem in pregnancy. The percentage of tests is even lower for the GBS swab, which is performed in only 66% of cases. A possible explanation is that this practice has only recently found some general implementation in our local reality.

Seroconversion rates and neonatal infections are rare, as shown by our data: no seroconversions were observed for Toxoplasma gondii, 4 seroconversions for CMV. In some cases with dubious patterns or with probable persistence of IgM, we had to perform additional diagnostic tests and follow-up.

The incidence of Toxoplasma infection in our sample was 0.12% (1/846), which is in good accord with the literature [24].

A test for CMV DNA in the urine of the newborn, which is a very simple way to immediately diagnose a congenital infection, was performed in all cases of seroconversion and in all dubious cases. We found two neonatal infections, both asymptomatic: one in a newborn whose mother had presented a seroconversion during pregnancy and another in a newborn whose mother had IgM and IgG positivity at first test in pregnancy.

According to those numbers, the prevalence of neonatal infection would be 0.25% (2/797). However, asymptomatic infections from “immune” mothers cannot be diagnosed by normal procedures. Since, according to some studies, these may represent more than half of the total infections, the true prevalence of CMV infection in our population could be about 0.5%, or even higher, in agreement with values in the literature [25].

Finally, although intrapartum antibiotic prophylaxis was not consistently performed in all swab positive cases, no GBS neonatal sepsis could be demonstrated in our population.

## Conclusions

In conclusion, even if congenital infection from Toxoplasma and CMV is not a very common event, a reliable immunological evaluation of the mother during pregnancy is certainly the best way to identify cases at risk.

Although our data are the result of a limited analysis, considering only a relatively small number of pregnancies and newborns, we think that they can be useful to provide some epidemiological information about our local situation, and to give an outline of the perinatal condition of risk for some congenital infections at our institution. It will certainly be useful to collect further data in order to confirm the values we reported.

## Competing interests

The authors declare that they have no competing interests.

## Authors’ contributions

GP made substantial contributions to conception and design of the study, and to analysis and interpretation of data, was involved in drafting the manuscript and revising it. CC made substantial contributions to conception and design of the study, was involved in drafting the manuscript and revising it. LAC made substantial contributions to the design of the study, to acquisition of data and to analysis and interpretation of data, was involved in drafting the manuscript. LC made substantial contributions to the design of the study, to acquisition of data and to analysis and interpretation of data, was involved in drafting the manuscript. AR made substantial contributions to acquisition and interpretation of data, was involved in drafting the manuscript. MGS made substantial contributions to acquisition and interpretation of data, was involved in drafting the manuscript. MG made substantial contributions to acquisition and interpretation of data, was involved in drafting the manuscript. GC made substantial contributions to conception and design of the study, was involved in drafting the manuscript and revising it critically for important intellectual content. All authors read and approved the final manuscript.
